# Study on Quality and Starch Characteristics of Powdery and Crispy Lotus Roots

**DOI:** 10.3390/foods13203335

**Published:** 2024-10-21

**Authors:** Zichen Cai, Yaying Jiang, Fei Wang, Jun Liu, Juan Kan, Man Zhang, Xiaohua Qi, Liangjun Li, Shuping Zhao, Chunlu Qian

**Affiliations:** 1 Department of Food Science and Engineering, School of Food Science and Engineering, Yangzhou University, Yangzhou 225127, China; m15370571156@163.com (Z.C.); jiangyaying761@163.com (Y.J.); wfei1101@163.com (F.W.); junliu@yzu.edu.cn (J.L.); kanjuan@yzu.edu.cn (J.K.); mzhang100@yzu.edu.cn (M.Z.); 2 Department of Horticulture, College of Horticulture and Landscape Architecture, Yangzhou University, Yangzhou 225009, China; xhqi@yzu.edu.cn (X.Q.); ljli@yzu.edu.cn (L.L.); zhaoshuping@yzu.edu.cn (S.Z.)

**Keywords:** lotus root, starch, heat processing, structural characteristics

## Abstract

Nine varieties of lotus root (*Suining*, *Xinhe*, *Zaohua*, *Zhonghua*, *L0014*, *L0013*, *Cuiyu*, *L0011*, and *Zhenzhu*) were selected as the research materials to compare their differences in physical, chemical, and starch characteristics before and after boiling, frying, and microwaving. The results showed that *Zhenzhu*, *Xinhe*, *L0013*, *Cuiyu*, and *Zhonghua* belong to the crispy lotus root type, while *L0011*, *L0014*, *Zaohua*, and *Suining* belong to the powdery lotus root type. Furthermore, the nine varieties were characterized for their starch by optical micrograph (OM), polarized micrograph (PM), scanning electron micrograph (SEM), attenuated total reflection Fourier-transform infrared spectroscopy (ATR-FTIR), X-ray diffraction (XRD), carbon-13 cross-polarization/magic angle spinning nuclear magnetic resonance (^13^C CP/MAS NMR), and differential scanning calorimetry (DSC). The starch granule of powdery lotus root appeared to be larger than that of crispy lotus, and ATR-FTIR studies revealed that the outer layer of starch granules from nine different varieties of lotus root had a highly organized structure. Moreover, XRD and ^13^C CP/MAS NMR analyses revealed that starch from eight lotus varieties (*Suining*, *Xinhe*, *Zaohua*, *Zhonghua*, *L0014*, *L0013*, *Cuiyu*, *L0011*) belong to the A-crystal type, while starch from *Zhenzhu* belongs to the C_A_-crystal type. The starch from powdery lotus root exhibited higher crystallinity, as well as increased gelatinization temperature and enthalpy, indicating that its crystal structure was relatively superior compared to that of crispy lotus starch. The short-range order degree, crystallinity, gelatinization temperature, and heat enthalpy of lotus starch decreased after boiling and frying but increased to varying extents after microwaving. Additionally, the heat resistance and stability of starch particles from crispy lotus root were improved after microwave treatment.

## 1. Introduction

The lotus root (*Nelumbo nucifera* Gaertn) is an aquatic plant belonging to the Nelumbonaceae family and is considered one of the oldest dicotyledonous plants [1]. In Asia, particularly in China, India, Korea, Thailand, and Japan, lotus root is highly popular and holds significant economic and agricultural value [2]. It is the underground part of lotus rhizomes, formed while being immersed in water, and possesses unique characteristics. The lotus root can develop multiple rhizomes in a single growing season, each with an average length of 10–20 cm, which produce and store a substantial amount of nutrients, particularly starch [3]. The starch content constitutes 10–20% of the total fresh weight of lotus root, varying among different varieties. Starch content and its characteristics are key factors in determining the storage and processing quality of lotus root. Significant differences exist in the quality characteristics of lotus root among different varieties, which can lead to inaccuracies in selecting appropriate varieties for cooking or processing lotus root products. Based on texture, lotus root can be classified into two types. The first type is crispy lotus root, characterized by high water and sugar content, low starch content, and a crispy, tender texture, making it suitable for use in cold dishes or stir-fried dishes. Crispy lotus root can also be processed into juice beverages, frozen lotus root, and prefabricated cold dishes. The starch from crispy lotus root has low viscosity, which makes it less prone to precipitation, with a high gelatinization temperature that contributes to its crispy and tender texture. The second type is powdery lotus root, which is characterized by a high starch content and low water content. The high starch content results in a soft texture with high viscosity after cooking [4]. Powdery lotus root can be processed into various products, such as lotus root powder and lotus root soup. After processing, powdery lotus root exhibits a soft texture, high amylopectin content, strong adhesive properties, and good starch stability. It is more suitable for hot processing methods such as boiling, steaming, and stewing.

This paper investigated the characteristics of nine representative varieties of lotus root and their starches and analyzed the changes in these characteristics after hot processing (boiling, frying, and microwaving). The results provide a theoretical basis for selecting suitable lotus root varieties for different cooking or processing methods and contribute to expanding the knowledge of the starch characteristics of lotus root.

## 2. Materials and Methods

### 2.1. Raw Materials

Nine varieties of lotus *Suining*, *Xinhe*, *Zaohua*, *Zhonghua*, *L0014*, *L0013*, *Cuiyu*, *L0011*, and *Zhenzhu* were harvested from the Aquatic Vegetable Resource Garden of Yangzhou University and transported to the laboratory within 2 h. Samples with uniform size, no mechanical damage, and no pest or disease injury were selected for experiments.

### 2.2. Separation of Lotus Root Starch

Fresh lotus root was washed, peeled, and sliced, and then soaked for 0.5 h for color protection using a solution containing 1% NaCl and 0.2% NaHSO_3_. The sliced lotus root was homogenized, mixed with water repeatedly, and filtered five times to collect the precipitate. The resulting starch was washed with clean water about five times, followed by washing with a 0.05% NaOH solution three times to deproteinize it. It was then washed with 85% ethanol three times to degrease. Finally, the purified starch was dried at 40 °C for 12 h, resulting in a dry, powdery form. The starch was then sieved through a 100-mesh sieve to obtain lotus root starch.

### 2.3. Methods Used for Starch Processing

The water boiling procedure was based on a previously described experimental method with slight modifications [5]. An amount of 5.0 g of accurately weighed dry lotus root starch was dispersed in 50 mL of water and stirred for 5 min. The sample was then heated in hot water at 100 °C for 10 min. After cooling, the sample was freeze-dried for 24 h, crushed, and sieved for later use.

The frying procedure was based on a previously described experimental method with slight modifications [6]. First, lotus root starch samples with a moisture content of 20% were prepared. Then, 5.0 g of lotus root starch was dispersed in 50 mL of soybean oil. After magnetic stirring for 5 min, the samples were fried in an oil bath at 180 °C for 5 min. The fried samples were then degreased, dried, ground, and passed through a 100-mesh sieve for later use.

The microwave procedure was based on a previously described research method with slight modifications [7]. First, lotus root starch samples with a moisture content of 20% were prepared. Then, 5.0 g of lotus root starch was weighed, and the samples were microwaved at 300 W for 5 min. Afterward, the samples were dried, ground, and passed through a 100-mesh sieve for later use.

### 2.4. Sensory Evaluation

The washed lotus root was peeled and cut into 1 cm-thick discs using a slicer. The slices were then boiled in water for 20 min and allowed to cool at room temperature. A total of 20 students from the laboratory, all experienced in food sensory evaluation, were invited to assess the quality of the nine varieties of lotus root. The sensory evaluation experiment was performed in complicance with the laws of the People’s Republic of China and institutional guidelines of Yangzhou University. The experiment also was approved by the Ethics Committee of Yangzhou University. All volunteers were mentally and physically able to participate in the sensory evaluation and an informed written consent was obtained from each volunteer.

### 2.5. Chromaticity Value Determination

A hand-held colorimeter was used to measure the chromaticity L*, a*, and b* values of the lotus root surface. The white board was used as a control to measure the changes in L*, a*, and b* values in the cross-section of the middle part of the lotus root, and replicated 3 times for each group. The E* value was calculated as $E^{*}=\sqrt{{\left(L-L^{*}\right)}^{2}+{\left(a-a^{*}\right)}^{2}+{\left(b-b^{*}\right)}^{2}}$, where L, a, and b were the color parameters of the standard white plate, and L*, a*, and b* were the color parameters of the lotus root.

### 2.6. Texture Profile Analysis (TPA)

The texture of fresh lotus root was determined using a texture analyzer (TMS-Pro, FTC, Atlanta, GA, USA), based on a previously described experimental method with slight modifications [8]. The middle part of the lotus root was cut into a cylinder (5 cm high), and the TPA was applied to the cross-section of the cylinder. The parameters of the texture analyzer were set as follows: the probe diameter was 5 mm, the pre-test and post-test speeds were 60.00 mm/s, the maximum induction force was 0.7 N, the compression ratio was 35%, and the sample was recompressed after holding for 5 s.

### 2.7. Determination of Quality Indices

Determination of moisture: the water content of the lotus root was determined by the GB method (GB 5009.3-2016) [9].

Determination of soluble sugar content: according to the method of Huang [10], the anthrone–sulfuric acid method was used to determine the content of soluble sugar.

Determination of titratable acid content: according to Caric’s method [11], the content of titratable acid was determined by phenolphthalein titration.

Determination of vitamin C (VC) content: according to the method of Kek [12], the content of VC was determined by indophenol titration.

Determination of soluble protein content: according to the method proposed by Bradford [13], the soluble protein content was determined by the Coomas bright blue G-250 method.

Determination of polyphenol content: according to Singleton’s method [14], the polyphenol content was determined by the Folin phenol method.

Determination of starch and amylose content: the starch and amylose content kit of Suzhou Keming Biological Co., Ltd., Suzhou, China, was used for determination.

### 2.8. Optical Microscopic Image of Lotus Root Starch

The procedure was based on a previously described method with slight modifications [15]. A total of 100 μL of distilled water was dropped onto a slide, and a trace amount of lotus root starch was mixed with it. The mixture was then covered with a coverslip, and a polarizing microscope (BA310Pol, Beijing Motic, Beijing, China) was used at 40 × 10 magnification to observe the optical (OM) and polarized (PM) microscopic images of the starch.

### 2.9. Scanning Electron Micrographs (SEM) of Lotus Root Starch

According to the previously described method [16], an scanning electron microscope (S-4800, Hitachi, Tokyo, Japan) was used for observation. The starch sample was mounted onto an aluminum platform and then sputter-coated with gold. Microscopic observation and photography of each sample were performed at an accelerating voltage of 15 kV and a magnification of 400×.

### 2.10. Attenuated Total Reflection Fourier-Transform Infrared Spectroscopy (ATR-FTIR) of Lotus Root Starch

According to the previously described experimental method [17], an FTIR spectrometer equipped with an ATR device was used for determination. Spectra were corrected from 1200 to 800 cm^−1^ using a baseline correction and processed with OMNIC 8.0 deconvolution. The band intensity ratios of 1045/1022 cm^−1^ and 1022/995 cm^−1^ were calculated.

### 2.11. X-Ray Diffraction (XRD) of Lotus Root Starch

According to the previously described experimental method [18], an X-ray diffractometer was used for analysis. The diffractometer operated at 40 kV and 40 mA, with nickel filtering for copper Kα radiation. The scanning range was set from 3° to 40° (2 min), with a scanning speed of 0.3°/θ. The relative crystallinity was measured following the previously described method [19].

### 2.12. Carbon-13 Cross-Polarization/Magic Angle Spinning Nuclear Magnetic Resonance (^13^C CP/MAS NMR) Spectrum of Lotus Root Starch

According to the previously described method [20], ^13^C CP/MAS NMR analysis of lotus root starch was performed using a spectrometer (Avance III 400WB, Bruker, Bilerica, MA, USA) at B_0_ = 9.4 T. Deconvolution of the NMR spectra was performed using PeakFitTM™ Version 4.12.

### 2.13. Thermal Properties of Lotus Root Starch

Following the previously described method [21], the thermal properties were determined using differential scanning calorimetry (DSC) (DSC 8500, PerkinElmer, Waltham, MA, USA). A starch sample (5 mg) was mixed with deionized water (15 μL), sealed in an aluminum pan, and equilibrated at room temperature for 12 h. The sample was then heated from 30 °C to 110 °C at a rate of 10 °C/min.

### 2.14. Swelling Power and Solubility of Lotus Root Starch

The previously described method was used with slight modifications [22]. A mixture of 0.1 g of starch and 10 mL of distilled water was placed in a water bath at 95 °C for 30 min for determination.

Solubility (SA) = Weight of water-soluble starch/weight of starch sample (dry) × 100%

Swelling power (SP) = Weight of expanded starch/[weight of starch sample (dry) × (100 − solubility)] × 100%

### 2.15. Data Analysis

SPSS 26.0 statistical software was used for the analysis of variance, and the Tukey method was applied to determine the significance of differences at *p* < 0.05.

## 3. Results

### 3.1. Sensory Evaluation of Lotus Root

**Figure 1:**
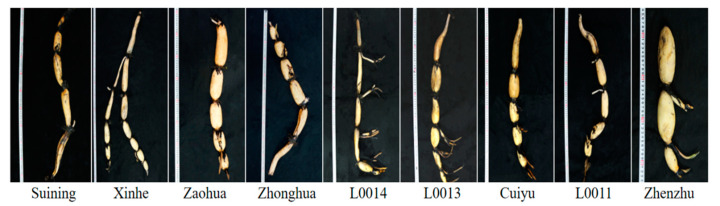
The phenotype of nine varieties of lotus root.

Through the sensory evaluation experiment (Figure 1), differences were observed in the appearance of the nine varieties of lotus root. The lotus nodes of *Suining*, *L0014*, and *L0011* were elongated and elliptical, while the lotus roots of *Zaohua* and *Zhenzhu* had larger nodes that were round and full. The lotus nodes of *Xinhe*, *Zhonghua*, *Cuiyu*, and *L0013* were slender, elliptical, and plump.

It was found that *Zhenzhu*, *Xinhe*, *L0013*, *Cuiyu*, and *Zhonghua* had a hard texture, high moisture content, and low starch content, and were classified as crispy lotus. In contrast, *Suining*, *Zaohua*, *L0014*, and *L0011* had a soft texture, low water content, and high starch content, and were classified as powdery lotus. The subsequent data on color (Table 1), texture (Table 2), and nutrient content (Table 3) of the lotus roots also confirmed this evaluation.

### 3.2. Determination of the Chromaticity Value of Lotus Root

The L* values of all lotus root varieties were above 50, with *Zhenzhu* and *Xinhe* showing higher values of 70.36 and 64.37, respectively (Table 1). The L* values of *L0013* and *Cuiyu* were smaller, at 53.45 and 57.58, respectively. All nine varieties of lotus root exhibited positive values for a* and b*, indicating that the skin color was reddish and yellowish. Among them, *Zhenzhu*, *Zaohua*, and *L0014* had higher a* values, at 8.04, 6.71, and 5.10, respectively. The lotus root E* values ranged from 21.15 to 34.37, and *Zhenzhu* showed the lowest E* value.

### 3.3. Texture Determination of Lotus Root

The textural characteristics of the nine lotus root varieties are presented in Table 2. *Suining*, *Zaohua*, and *L0014* exhibited relatively higher hardness, with values of 427.12, 393.87, and 328.36 N, respectively. The cohesion values for *Suining*, *Zaohua*, and *L0014* were 0.46, 0.40, and 0.33, respectively. *Cuiyu*, *L0011*, and *Suining* demonstrated the greatest elasticity, measuring 2.12, 1.94, and 1.55 mm, respectively. *Suining*, *Zaohua*, and *L0014* also had higher gumminess values, at 98.43, 91.57, and 91.57 N, respectively.

### 3.4. Nutrient Content of Different Varieties of Lotus Root

The moisture content of the nine lotus root varieties ranged from 67.17% to 82.99% (Table 3). The moisture content in the crispy lotus varieties *Zhenzhu*, *Xinhe*, *L0013*, *Cuiyu*, and *Zhonghua* was higher than that in the powdery lotus varieties *Suining*, *Zaohua*, *L0014*, and *L0011*. The soluble sugar and titratable acid contents of the nine lotus varieties ranged from 7.33 to 43.52 mg/g and 0.15% to 0.49%, respectively. The highest soluble sugar contents were found in two crispy lotus varieties, *Zhenzhu* and *Cuiyu*, with values of 43.52 and 30.96 mg/g, respectively. The sugar-to-acid ratio in crispy lotus root was relatively higher than in powdery lotus root, resulting in a crisper and sweeter taste.

The VC content in lotus root ranged from 32.09 to 57.13 mg/100 g, with the highest content observed in the crispy variety *L0013*. The phenolic content in the nine lotus varieties fluctuated between 13.66 and 24.09 mg/g, with the lowest phenolic content found in the crispy lotus variety *Zhenzhu* (13.66 mg/g). The protein content of both powdery and crispy lotus root varieties showed similar characteristics. Starch content across the nine varieties ranged from 7.53% to 14.46%, while amylose content ranged from 28.86% to 36.75%. The starch content of crispy lotus root was noticeably lower than that of powdery lotus root. The ratio of amylose to total starch was lower in powdery lotus root compared to crispy lotus root, while the ratio of amylopectin to total starch was higher in powdery lotus root. Overall, the general nutrient content of powdery lotus root was higher than that of crispy lotus root.

### 3.5. Correlation Analysis of Nutrient Component Content in Lotus Root

**Figure 2:**
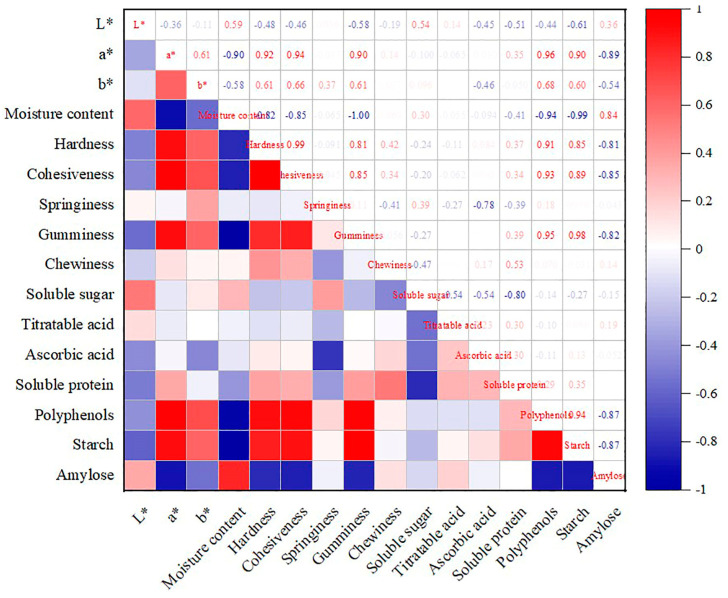
Pearson correlation analysis of nutritional components of different varieties of lotus root.

Correlation analysis revealed that the moisture content of lotus root had a significant negative correlation with starch content, polyphenol content, stickiness, hardness, and the a* value, with correlation coefficients of −0.97, −0.94, −1.00, −0.82, and −0.90, respectively (Figure 2). Hardness was positively correlated with starch content, polyphenol content, and a* value, with correlation coefficients of 0.85, 0.91, and 0.81 (Figure 2).

### 3.6. Optical Microscopy of Lotus Root Starch

**Figure 3:**
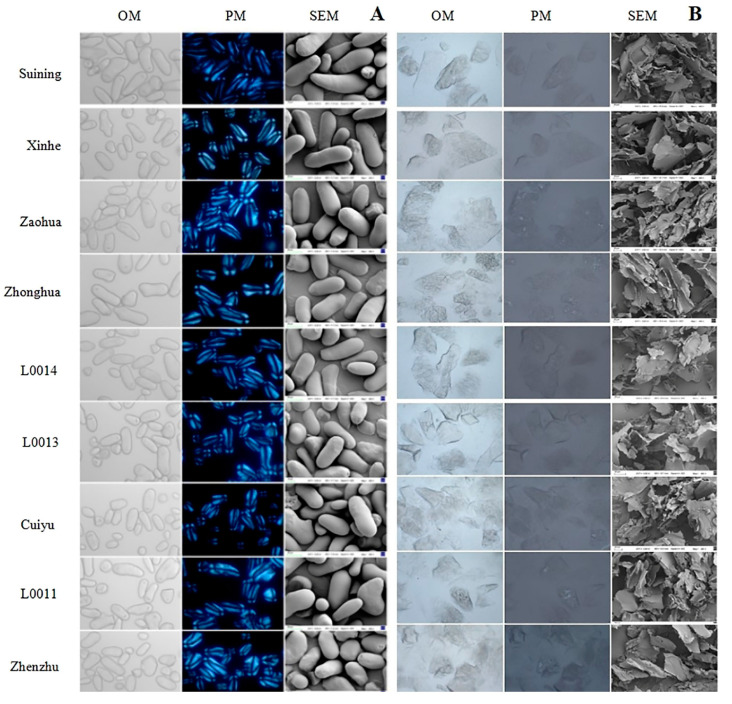

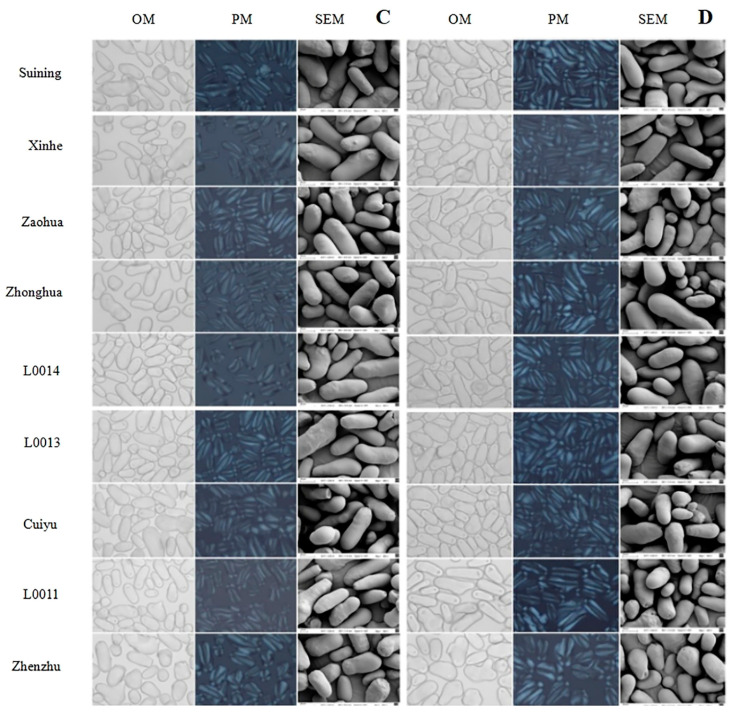
Microscopic observation of starch from different varieties of lotus root. (**A**) Optical microscope (OM), polarizing microscope (PM), and scanning electron microscope (SEM) images of raw starch from nine varieties of lotus root. (**B**) Optical microscope (OM), polarizing microscope (PM), and scanning electron microscope (SEM) images of boiled starch from nine varieties of lotus root. (**C**) Optical microscope (OM), polarizing microscope (PM), and scanning electron microscope (SEM) images of fried starch from nine varieties of lotus root. (**D**) Optical microscope (OM), polarizing microscope (PM), and scanning electron microscope (SEM) images of microwaved starch from nine varieties of lotus root.

The surface of all raw starch particles from lotus root was smooth, without cracks, and appeared elongated and oval, with varying sizes (Figure 3A). The umbilical region and birefringence on the starch surface were clearly visible. Through scanning electron microscopy, the starch particle sizes of *Suining*, *Zaohua*, *L0014*, and *L0011* were approximately 60 μm, and the particles were large and intact. In contrast, for the crispy lotus root varieties *Zhenzhu*, *Xinhe*, *L0013*, *Cuiyu*, and *Zhonghua*, the particle sizes ranged from 44 to 55 μm, with a higher proportion of smaller, irregularly shaped particles.

As shown in Figure 3B, all starch particles from the lotus root were completely disintegrated after boiling, with the particles swelling and gelatinizing as they absorbed water. Under the optical microscope, the starch appeared as a lumpy gel mass. In the polarizing microscope, the polarized structure of the starch disappeared and showed no birefringence. Scanning electron microscopy revealed fragmented flakes, indicating that the starch structure had been destroyed.

As shown in Figure 3C,D, after frying and microwaving, the starch particles from lotus roots exhibited a depression at the navel on their surface under the optical microscope, with the depression in fried starch being more pronounced than in microwaved starch. Additionally, the surface of the fried starch samples showed slight damage. Under the polarizing microscope, the polarized structure of both fried and microwaved starch did not completely disappear, but the birefringence was weakened. Scanning electron microscopy showed that the starch particles remained intact, but surface cracks were visible. The number of cracks was greater in fried starch particles than in microwaved ones, and crispy lotus starch had significantly more cracks than powdery lotus starch after both frying and microwaving.

### 3.7. Infrared Spectrum of Lotus Root Starch

**Figure 4:**
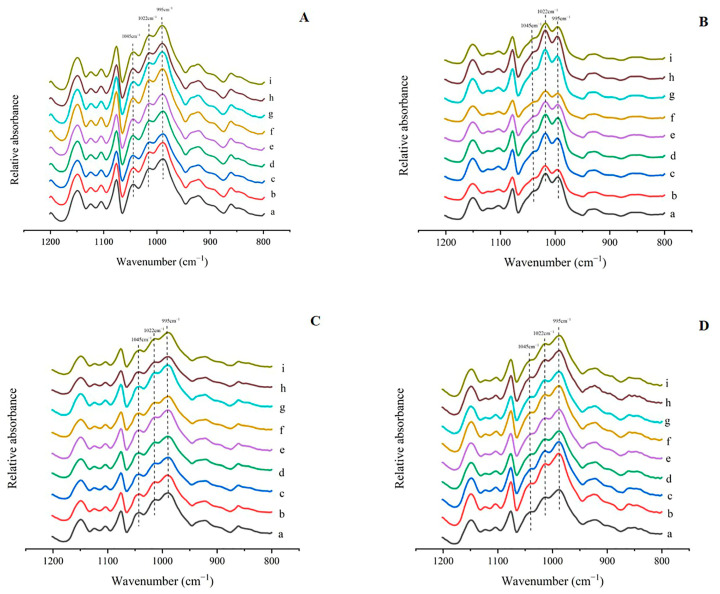
Infrared spectra of starch from nine varieties of lotus root. (**A**) Infrared spectra of raw starch from nine varieties of lotus root. (**B**) Infrared spectra of starch from nine varieties of lotus root after boiling. (**C**) Infrared spectra of starch from nine varieties of lotus root after frying. (**D**) Infrared spectra of starch from nine varieties of lotus root after microwave treatment. (a) Suining, (b) Xinhe, (c) Zaohua, (d) Zhonghua, (e) L0014, (f) L0013, (g) Cuiyu, (h) L0011, and (i) Zhenzhu.

The infrared spectra of starch from all lotus root varieties were similar (Figure 4). As shown in Table 4, the R1 and R2 ratios ranged from 0.74 to 0.77 and 0.73 to 0.75, respectively, with no significant differences observed in these ratios. After boiling, the R1 and R2 ratios for all lotus root starch samples were distributed between 1.06 and 1.12 and between 0.63 and 0.67, respectively. Following frying, the R1 and R2 ratios fluctuated between 0.70 and 0.72 and between 0.78 and 0.80, respectively.

Compared to raw starch, the absorbance ratio at 1022/995 cm^−1^ of lotus starch increased after frying, while the absorbance ratio at 1045/1022 cm^−1^ decreased. After microwaving, the R1 and R2 ratios of all lotus root starches changed to 0.71–0.74 and 0.77–0.83, respectively. In comparison to raw starch, the 1022/995 cm^−1^ absorbance ratio of microwaved starch decreased, while the 1045/1022 cm^−1^ absorbance ratio increased. The degree of order for all lotus root starch after microwaving was slightly higher than that after frying.

### 3.8. X-Ray Diffraction of Lotus Root Starch

**Figure 5:**
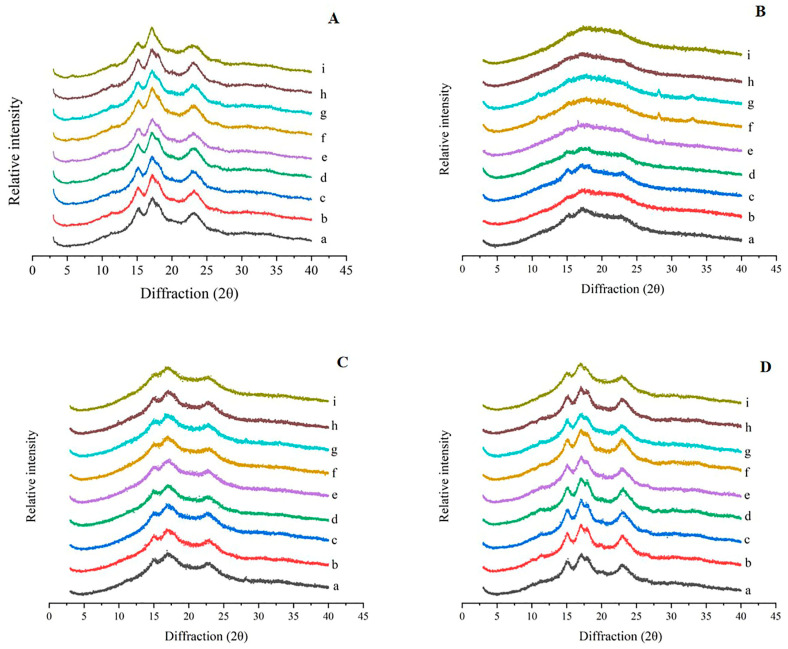
X-ray diffraction of starch from nine varieties of lotus root. (**A**) XRD diffraction patterns of raw starch from nine varieties of lotus root. (**B**) XRD diffraction patterns of starch from nine varieties of lotus root after boiling. (**C**) XRD diffraction patterns of starch from nine varieties of lotus root after frying. (**D**) XRD diffraction patterns of starch from nine varieties of lotus root after microwave treatment. (a) Suining, (b) Xinhe, (c) Zaohua, (d) Zhonghua, (e) L0014, (f) L0013, (g) Cuiyu, (h) L0011, and (i) Zhenzhu.

The XRD diffraction pattern of starch from Zhenzhu displayed a small peak at 5.6° 2θ and strong diffraction peaks at 15°, 17°, and 23° 2θ, characteristic of C-type crystals (closer to C_A_-type). Starch from the other eight varieties of lotus root exhibited strong diffraction peaks and unresolved double peaks at 17° and 18° 2θ, which correspond to A-type crystal forms (Figure 5A). The crystallinity of starch from the nine lotus root varieties ranged from 28.0% to 34.3% (Table 4). The powdery lotus root varieties Suining, Zaohua, L0014, and L0011 had higher crystallinity (34.3%, 33.5%, 32.1%, and 31.9%, respectively). In contrast, the crispy lotus root varieties Zhenzhu, Xinhe, and L0013 had lower crystallinity (28.0%, 28.5%, and 29.3%, respectively).

After boiling, the main diffraction peaks of starch from all lotus root varieties nearly disappeared, and the crystallinity decreased significantly (Figure 5B, Table 4). As shown in Figure 5C, after frying, lotus root starch exhibited strong diffraction peaks at 15°, 17°, and 23° 2θ, with the double peaks at 17° and 18° 2θ merging into a single peak, indicative of A-type crystals. Similarly, after microwaving (Figure 5D), lotus root starch showed strong diffraction peaks at 15° and 23° 2θ, with an unresolved doublet at 17° and 18° 2θ, which is also characteristic of A-type crystals.

After frying and microwaving, the crystal type of Zhenzhu starch shifted from C_A_-type to A-type. The crystal form of starch from the other eight lotus root varieties remained unchanged after frying and microwaving. Following frying, the diffraction peak intensity and relative crystallinity of lotus root starch decreased, with crystallinity ranging from 16.1% to 19.5%. The relative crystallinity of powdery lotus starch remained higher than that of crispy lotus root starch. After microwaving, the intensity of diffraction peaks did not change significantly, and the crystallinity increased slightly, ranging from 30.1% to 34.5%. The crystallinity of starch from crispy lotus root was relatively higher than that of powdery lotus root after microwaving.

### 3.9. ^13^C CP-MAS NMR Spectra of Lotus Root Starch

**Figure 6:**
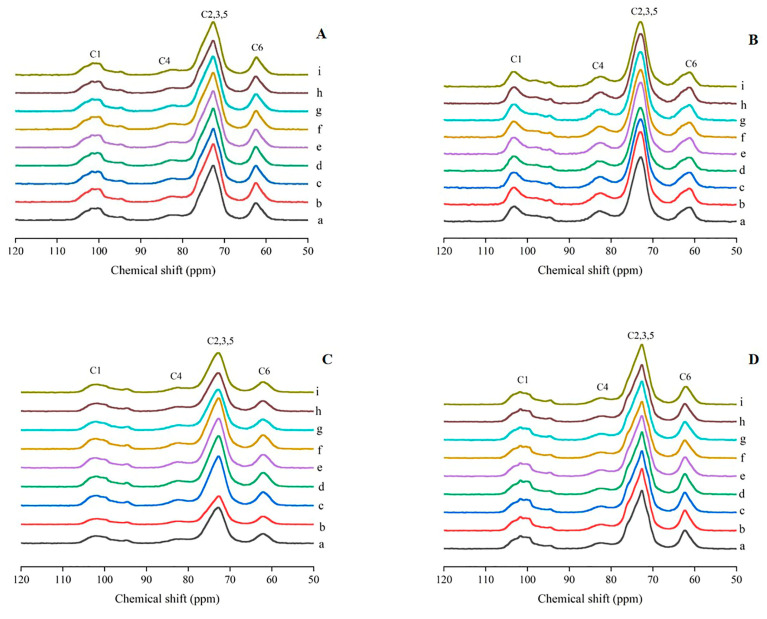
^13^C CP-MAS NMR spectra of starch from nine varieties of lotus root. (**A**) ^13^C CP-MAS NMR spectra of raw starch from nine varieties of lotus root. (**B**) ^13^C CP-MAS NMR spectra of starch from nine varieties of lotus root after boiling. (**C**) ^13^C CP-MAS NMR spectra of starch from nine varieties of lotus root after frying. (**D**) ^13^C CP-MAS NMR spectra of starch from nine varieties of lotus root after microwave treatment. (a) Suining, (b) Xinhe, (c) Zaohua, (d) Zhonghua, (e) L0014, (f) L0013, (g) Cuiyu, (h) L0011, and (i) Zhenzhu.

As shown in Figure 6A, the NMR spectra of starch from *Zhenzhu* displayed double peaks at 100.8 and 101.6 ppm in the C1 resonance region, indicating that the filler type was close to the C_A_-type. In contrast, the NMR spectra of starch from the other eight lotus root varieties did not show obvious triple peaks (at 101.6, 100.2, and 99.1 ppm), suggesting that the packing types of the starch granules in these varieties were similar to type A. After boiling, the double peaks in the C1 region of *Zhenzhu* starch disappeared, while triple peaks appeared in the C1 region of starch from the other eight varieties of lotus root (Figure 6B).

As shown in Figure 6C, no obvious triple peaks were observed in the lotus starch at 99.73, 100.57, and 101.68 ppm after frying, indicating that the filler type of all starch granules after frying was similar to type A. After microwaving, all starch samples exhibited three peaks at 99.73, 100.47, and 101.91 ppm, indicating that the filler type of starch granules remained similar to type A. These results were consistent with the XRD findings (Figure 6D).

### 3.10. Thermal Properties of Lotus Root Starch

The gelatinization parameters initial gelatinization temperature (T_0_), peak gelatinization temperature (T_p_), end gelatinization temperature (T_c_), and enthalpy (ΔH) for raw lotus root starch ranged from 61.01 to 66.9 °C, 64.76 to 71.25 °C, 67.98 to 80.51 °C, and 9.42 to 13.75 J/g, respectively. The T_0_ of starch from *L0011*, *Zhonghua*, *Zaohua*, and *L0014* was significantly (*p* < 0.05) higher than that of the other varieties, with values of 66.54 °C, 66.15 °C, 65.90 °C, and 64.62 °C, respectively (Table 5). Additionally, for the T_p_, starch from *Zhonghua*, *Zaohua*, *L0011*, and *L0014* also showed significantly higher values (*p* < 0.05) than the other varieties, at 71.25 °C, 71.06 °C, 69.73 °C, and 69.11 °C, respectively. During gelatinization, the ΔH of starch from the powdery lotus varieties *Suining*, *Zaohua*, *L0014*, and *L0011* was higher, at 13.75 J/g, 12.12 J/g, 11.64 J/g, and 11.49 J/g, respectively, while starch from the crispy lotus varieties *Zhenzhu*, *Xinhe*, *L0013*, and *Cuiyu* exhibited relatively lower gelatinization enthalpy.

Lotus starch was fully gelatinized after boiling, leaving no measurable gelatinization parameters. After frying, the T_0_ of lotus starch varied from 55.01 to 61.29 °C, the T_p_ ranged from 58.32 to 67.21 °C, the T_c_ ranged from 65.37 to 75.37 °C, and the ΔH varied from 6.13 to 11.49 J/g. Compared to raw starch, the T_0_, T_p_, T_c_, and ΔH of lotus root starch decreased after frying, with powdery lotus starch exhibiting significantly higher ΔH values than crispy lotus starch.

After microwaving, the T_0_ of lotus starch ranged from 64.36 to 70.29 °C, the T_p_ varied from 67.23 to 77.42 °C, the T_c_ ranged from 74.37 to 84.38 °C, and the ΔH ranged from 11.41 to 15.98 J/g. In comparison to raw starch, the T_0_, T_p_, T_c_, and ΔH of lotus starch increased after microwaving. Additionally, the ΔH increase in crispy lotus starch after microwaving was relatively higher than that in powdery lotus starch.

## 4. Discussion

Lotus root is the largest aquatic vegetable in China, and its quality is usually evaluated by appearance and texture [23], with further assessments focusing on flavor and nutrient components [24,25,26]. In this study, the nine varieties of lotus root exhibited distinct color and texture characteristics, as well as differences in sugar, acid, and phenolic content, despite being grown under the same conditions. These differences are likely due to the biological variations between the varieties. These findings are consistent with previous reports [27]. Notably, the total phenolic content of powdery lotus root was significantly higher than that of crispy lotus root, and the a* value of powdery lotus root was larger. This may be due to the higher phenolic content in powdery lotus root, as phenols can oxidize, leading to color changes.

Starch is the main component of lotus root and provides dietary energy for humans [28]. The starch and amylose contents of the nine varieties of lotus root were similar to previous reports [29,30], and the differences between varieties can also be attributed to biological factors.

After boiling, the starch particles of the lotus root were highly damaged and irregular in shape. Deep spots, roughness, and cracks appeared on the surface of starch particles after frying and microwaving. These results are consistent with earlier research [31,32,33]. Boiling completely destroyed the Maltese cross structure of lotus root starch, indicating that the internal crystal structure was disrupted and chemical bonds were broken [34]. Frying and microwaving altered the brightness of the Maltese cross, with black areas expanding, as thermal processing damaged the crystal layers of starch particles. Frying caused more severe damage to starch particles than microwaving, as indicated by weaker birefringence after frying. Additionally, the number of cracks and deep spots on the surface of crispy lotus starch particles after frying and microwaving was significantly higher than that of powdery lotus starch, suggesting that crispy lotus starch has lower stability.

After boiling and frying, the R1 value of starch from crispy lotus root decreased, primarily due to the higher water content and temperature during these processes, which caused the double helices in the outer regions of the starch particles to expand and be destroyed, reducing the short-range molecular order and R1 value. The R1 value of starch from powdery lotus root was higher than that of crispy lotus root, indicating that powdery lotus starch was more resistant to high temperatures. After microwaving, the R1 value increased by 0.03–0.1, likely due to microwave-induced molecular rearrangement in the starch crystallization region, promoting the formation of more ordered structures [7]. These findings are consistent with previous studies [35,36].

The R2 value reflects the hydration degree of the crystalline region within starch particles [37]. The R2 value of starch from lotus root increased after boiling and frying, indicating an increase in starch hydration. This may be due to gelatinization, which promotes the interaction between starch and water molecules, resulting in more water molecules bound to starch. The R2 value of starch from crispy lotus root was higher than that of powdery lotus root, suggesting that crispy lotus starch molecules more easily combine with water. After microwaving, the R2 value decreased, indicating that the interaction between starch and water was inhibited, and the hydration degree in the crystalline zone was lower, consistent with the findings of Cheng [38].

The short-range order detected by FTIR is mainly related to the level of double helices, while the long-range order detected by XRD is associated with the accumulation of double helices [6]. The characteristic XRD peaks of lotus root starch disappeared after boiling, indicating the destruction of the crystal structure and the formation of amorphous regions, as boiling breaks the double helices formed by amylose and amylopectin. Despite this, the crystallinity of boiled lotus root starch ranged from 4.6% to 9.5%, indicating that a small amount of crystallinity remained. The crystal structure of lotus root starch did not change after frying, but the double peaks at 17° and 18° 2θ merged into a single peak. The diffraction peak intensity and crystallinity of lotus root starch decreased after frying, likely due to the loss and melting of the crystalline regions caused by hydrogen bond disruption. The decrease in relative crystallinity may be due to the instability of the starch layer arrangement, and the double helix structure of the amylopectin crystal region became more fragile under frying conditions [39,40]. Similar changes have been observed in potato starch after frying [6].

After microwaving, there was no significant change in the intensity of the starch diffraction peak, and the starch from *Zhenzhu* transitioned from C_A_-type to A-type crystallization. This may be because C_A_-type crystals are a mix of A- and B-type starches, with B-type starches having a weaker crystal structure, looser double helix arrangement, and more water molecules between helices, making them more susceptible to microwave treatment [41]. After microwaving, the crystallinity of lotus root starch increased, likely due to the rearrangement of starch crystals, resulting in a more compact structure. Similar changes have been observed in rice starch after microwave treatment combined with annealing [42], and similar findings have been reported previously [43,44]. Additionally, after frying and microwaving, the crystallinity of powdery lotus starch was significantly higher than that of crispy lotus starch, indicating that the crystal structure of powdery lotus starch is more resistant to thermal damage.

^13^C CP-MAS NMR is a powerful technique for studying starch helical conformation and carbonyl shift. The C1 and C4 peaks are related to the ordered and amorphous components of starch, respectively [45,46]. The peak at 103 ppm in the C1 region reflects the amorphous state of starch. After boiling, the triple peak in the C1 region of lotus root starch disappeared, and a single peak appeared at 103 ppm, indicating that the starch had transitioned into an amorphous state after gelatinization. The signal intensity of the C4 peak increased after boiling, indicating an increase in the non-crystalline content of starch, which was consistent with the XRD results. After frying, the triple peak signal in the C1 region of all starch samples weakened, likely due to the increase in the amorphous region and decrease in the crystalline region caused by high temperatures, consistent with FTIR and XRD results. After microwaving, no new peaks appeared in the NMR spectrum of lotus root starch, and the signal intensity in the C1 region increased slightly, a phenomenon also reflected in the FTIR and XRD results. The helical content of starch in indica rice increased after microwaving, with a corresponding increase in order and crystallinity [47], similar to the results of this study. After microwaving, the three peaks in the C1 region of starch from all nine lotus root varieties were characteristic of A-type crystals, consistent with the XRD findings.

Starch gelatinization is influenced by amylopectin, amylose content, and the ratio of crystalline to amorphous regions [48]. After boiling, the starch samples were fully gelatinized, with their grain and crystal structures destroyed, resulting in the disappearance of the gelatinization peak and no measurable gelatinization parameters. The gelatinization temperature and ΔH of lotus root starch after frying were significantly lower than those of raw starch, indicating that gelatinization occurred during frying. Frying damaged the helical structure of starch and reduced its thermal stability, so less energy and a lower temperature were required to melt the double helices. These findings are consistent with FTIR and XRD results, and previous studies by Wang [49]. Additionally, the ΔH of gelatinization after frying was higher than that after boiling, indicating that more energy is required to destroy the crystalline regions of a more stable starch structure. The gelatinization temperature and ΔH of lotus root starch after microwaving were significantly higher than those of raw starch, suggesting that the double helix rearrangement of starch molecules during microwaving increased the density and stability of the crystals, requiring higher temperatures for complete gelatinization [50]. Furthermore, the increase in gelatinization ΔH after microwaving was greater for crispy lotus starch than for powdery lotus starch, possibly due to the melting of weaker microcrystals in crispy lotus starch during microwaving, which made the starch molecules more stable [51].

## 5. Conclusions

Among the nine varieties of lotus root, five (*Zhenzhu*, *Xinhe*, *L0013*, *Cuiyu*, and *Zhonghua*) are classified as the crispy lotus root type, while four (*L0011*, *L0014*, *Zaohua*, and *Suining*) are classified as the powdery lotus root type. The color of powdery lotus root is more reddish, and its hardness, stickiness, and cohesion are significantly higher than those of crispy lotus root. Crispy lotus root, characterized by high moisture content, VC, and a high sugar-to-acid ratio, is suitable for cold dishes and stir-frying. In contrast, powdery lotus root, with higher polyphenol, protein, and starch contents, has a softer texture and is ideal for making soups. The starch from powdery lotus root exhibits higher crystallinity, gelatinization temperature, gelatinization enthalpy, expansibility, and a more stable molecular structure. On the other hand, the starch from crispy lotus root has lower crystallinity and poorer stability and heat resistance, but better gumminess, higher hardness, and viscosity. After boiling and frying, the short-range order, crystallinity, gelatinization temperature, and enthalpy of lotus root starch decreased, with powdery lotus root starch granules demonstrating good heat resistance and stability. Following microwave treatment, the short-range order, crystallinity, gelatinization temperature, and gelatinization enthalpy of lotus root starch increased to varying degrees. Notably, the heat resistance and stability of crispy lotus root starch granules improved after microwave treatment.

## Figures and Tables

**Table 1:** The color difference of nine varieties of lotus root.

	L*	a*	b*	Ε*
Suining	57.77 ± 1.23 ^d^	8.04 ± 0.45 ^a^	23.82 ± 0.55 ^a^	34.37 ± 1.57 ^a^
Xinhe	64.37 ± 1.14 ^e^	1.54 ± 0.13 ^e^	18.65 ± 1.11 ^cd^	25.26 ± 0.05 ^e^
Zaohua	58.73 ± 1.13 ^e^	6.71 ± 0.56 ^b^	18.99 ± 1.29 ^cd^	30.36 ± 1.45 ^bcd^
Zhonghua	58.66 ± 0.12 ^h^	3.99 ± 0.68 ^d^	18.62 ± 0.76 ^cd^	29.83 ± 2.56 ^cd^
L0014	59.15 ± 0.36 ^f^	5.10 ± 0.53 ^c^	23.05 ± 0.93 ^a^	32.32 ± 0.49 ^abc^
L0013	53.45 ± 1.54 ^b^	2.06 ± 0.34 ^e^	16.32 ± 0.69 ^e^	32.81 ± 1.19 ^ab^
Cuiyu	57.58 ± 0.24 ^g^	2.03 ± 0.11 ^e^	21.34 ± 0.45 ^b^	32.13 ± 0.82 ^abc^
L0011	62.55 ± 1.36 ^c^	4.78 ± 0.17 ^cd^	20.07 ± 0.29 ^bc^	27.81 ± 0.96 ^de^
Zhenzhu	70.36 ± 2.54 ^a^	1.16 ± 0.11 ^e^	18.32 ± 0.72 ^d^	21.15 ± 0.87 ^f^

Different subscript letters in the same row for the same item indicate significant (*p* < 0.05) differences.

**Table 2:** Texture characteristics of nine varieties of lotus root.

	Hardness (N)	Cohesiveness	Springiness	Gumminess (N)
Suining	427.12 ± 15.89 ^a^	0.46 ± 0.03 ^a^	1.55 ± 0.12 ^c^	98.43 ± 5.23 ^a^
Xinhe	219.77 ± 5.59 ^h^	0.14 ± 0.01 ^h^	1.46 ± 0.09 ^d^	32.00 ± 0.01 ^g^
Zaohua	393.87 ± 4.34 ^b^	0.40 ± 0.01 ^b^	1.36 ± 0.06 ^e^	91.57 ± 6.56 ^b^
Zhonghua	259.25 ± 5.19 ^d^	0.21 ± 0.01 ^de^	1.20 ± 0.16 ^g^	70.39 ± 5.73 ^d^
L0014	328.36 ± 9.02 ^c^	0.33 ± 0.01 ^c^	1.29 ± 0.24 ^f^	91.57 ± 1.98 ^b^
L0013	240.55 ± 12.43 ^f^	0.18 ± 0.02 ^e^	1.20 ± 0.11 ^g^	49.10 ± 3.03 ^f^
Cuiyu	255.93 ± 3.21 ^e^	0.21 ± 0.01 ^de^	2.12 ± 0.13 ^a^	58.02 ± 5.54 ^e^
L0011	236.88 ± 10.67 ^g^	0.21 ± 0.01 ^de^	1.94 ± 0.23 ^b^	85.70 ± 1.02 ^c^
Zhenzhu	182.55 ± 1.92 ^i^	0.11 ± 0.01 ^g^	1.37 ± 0.18 ^e^	9.22 ± 1.21 ^h^

Different subscript letters in the same row for the same item indicate significant (*p* < 0.05) differences.

**Table 3:** Nutrient content of different varieties of lotus root.

	Suining	Xinhe	Zaohua	Zhonghua	L0014	L0013	Cuiyu	L0011	Zhenzhu
Moisture content (%)	67.17 ± 0.31 ^g^	79.92 ± 0.99 ^b^	67.89 ± 0.19 ^fg^	72.5 ± 0.89 ^d^	68.01 ± 0.29 ^f^	75.15 ± 0.22 ^c^	75.04 ± 0.21 ^c^	69.32 ± 0.55 ^e^	82.99 ± 0.71 ^a^
Soluble sugar (mg/g)	29.38 ± 1.31 ^bc^	12.24 ± 0.58 ^f^	15.63 ± 0.97 ^e^	29.55 ± 1.45 ^bc^	7.33 ± 0.54 ^h^	7.43 ± 0.62 ^h^	30.96 ± 0.72 ^b^	28.82 ± 0.61 ^c^	43.52 ± 1.29 ^a^
Titratable acid (%)	0.20 ± 0.01 ^e^	0.31 ± 0.01 ^c^	0.28 ± 0.01 ^d^	0.14 ± 0.01 ^g^	0.49 ± 0.01 ^a^	0.35 ± 0.02 ^b^	0.17 ± 0.01 ^f^	0.34 ± 0.01 ^b^	0.32 ± 0.01 ^c^
Ascorbic acid (mg/100 g)	34.44 ± 0.18 ^g^	37.57 ± 0.61 ^e^	51.66 ± 0.94 ^b^	51.66 ± 1.10 ^b^	50.09 ± 0.32 ^c^	57.13 ± 0.98 ^a^	36.01 ± 0.29 ^f^	32.09 ± 0.99 ^h^	40.69 ± 0.19 ^d^
Soluble protein (mg/g)	3.19 ± 0.02 ^b^	3.28 ± 0.01 ^a^	3.11 ± 0.02 ^c^	2.99 ± 0.01 ^d^	3.13 ± 0.02 ^c^	3.32 ± 0.05 ^a^	2.70 ± 0.03 ^f^	3.06 ± 0.01 ^e^	2.61 ± 0.03 ^g^
Polyphenols (mg/g)	24.09 ± 0.23 ^a^	15.72 ± 0.17 ^e^	23.29 ± 0.31 ^b^	18.55 ± 0.28 ^d^	21.01 ± 0.21 ^c^	15.68 ± 0.32 ^e^	18.55 ± 0.52 ^d^	20.67 ± 0.29 ^c^	13.66 ± 0.17 ^f^
Starch (%)	15.46 ± 0.22 ^a^	8.98 ± 0.09 ^g^	13.06 ± 0.01 ^b^	11.49 ± 0.18 ^d^	13.03 ± 0.27 ^b^	8.92 ± 0.45 ^f^	10.05 ± 0.14 ^e^	12.76 ± 0.11 ^c^	7.53 ± 0.18 ^h^
Amylose (%)	21.86 ± 0.93 ^a^	34.21 ± 0.52 ^g^	29.43 ± 0.42 ^b^	31.73 ± 0.11 ^d^	29.53 ± 0.93 ^b^	32.74 ± 0.73 ^f^	32.12 ± 0.41 ^e^	30.70 ± 0.27 ^c^	36.75 ± 0.67 ^h^

Different subscript letters in the same row for the same item indicate significant (*p* < 0.05) differences.

**Table 4:** IR absorption ratio and crystallinity of starch from nine varieties of lotus root.

	FTIR Ratios								Relative Crystallinity (%)			
	Raw		Boiled		Fried		Microwave		Raw	Boiled	Fried	Microwave
	R1	R2	R1	R2	R1	R2	R1	R2				
Suining	0.75	0.73	0.65	1.06	0.70	0.79	0.74	0.77	34.3 ± 0.26 ^a^	9.5 ± 0.04 ^a^	19.5 ± 0.25 ^a^	34.5 ± 0.24 ^a^
Xinhe	0.76	0.74	0.66	1.10	0.71	0.78	0.73	0.82	28.5 ± 0.17 ^g^	4.9 ± 0.19 ^e^	17.2 ± 0.14 ^g^	30.2 ± 0.2 ^gh^
Zaohua	0.75	0.74	0.65	1.09	0.7	0.80	0.74	0.82	33.5 ± 0.34 ^b^	8.7 ± 0.18 ^b^	18.5 ± 0.27 ^b^	33.5 ± 0.54 ^b^
Zhonghua	0.77	0.74	0.64	1.05	0.71	0.80	0.74	0.83	30.9 ± 0.45 ^d^	6.4 ± 0.12 ^c^	17.6 ± 0.10 ^e^	31.6 ± 0.31 ^f^
L0014	0.75	0.75	0.66	1.05	0.71	0.79	0.71	0.83	32.1 ± 0.10 ^c^	6.5 ± 0.06 ^c^	18.2 ± 0.16 ^c^	33.2 ± 0.34 ^c^
L0013	0.75	0.73	0.67	1.06	0.71	0.79	0.72	0.83	29.3 ± 0.15 ^f^	5.5 ± 0.13 ^d^	17.3 ± 0.10 ^h^	30.3 ± 0.3 ^g^
Cuiyu	0.75	0.73	0.64	1.12	0.7	0.79	0.72	0.82	30.0 ± 0.14 ^e^	5.7 ± 011 ^d^	17.5 ± 0.20 ^f^	32.5 ± 0.15 ^d^
L0011	0.76	0.74	0.63	1.12	0.71	0.79	0.73	0.82	31.9 ± 0.13 ^c^	6.5 ± 0.16 ^c^	18 ± 0.11 ^d^	32.0 ± 0.2 ^e^
Zhenzhu	0.74	0.74	0.66	1.07	0.72	0.79	0.71	0.83	28.0 ± 0.18 ^h^	4.6 ± 0.18 ^f^	16.1 ± 0.10 ^i^	30.1 ± 0.26 ^i^

Different subscript letters in the same row for the same item indicate significant differences (*p* < 0.05). R1 (1045/1022 cm^−1^); R2 (1022/995 cm^−1^).

**Table 5:** Thermal properties of lotus root starch.

		Suining	Xinhe	Zaohua	Zhonghua	L0014	L0013	Cuiyu	L0011	Zhenzhu
Raw	T_0_/°C	62.85 ± 0.11 ^f^	64.29 ± 0.27 ^e^	65.9 ± 0.25 ^c^	66.15 ± 0.13 ^b^	64.62 ± 0.42 ^d^	62.11 ± 0.22 ^g^	62.01 ± 0.17 ^g^	66.54 ± 0.74 ^a^	61.01 ± 0.28 ^h^
	T_P_/°C	66.91 ± 0.19 ^f^	68.33 ± 0.20 ^e^	71.06 ± 0.15 ^b^	71.25 ± 0.10 ^a^	69.11 ± 0.21 ^d^	66.19 ± 0.28 ^g^	66.15 ± 0.13 ^g^	69.73 ± 0.22 ^c^	64.76 ± 0.26 ^h^
	T_c_/°C	73.32 ± 0.15 ^e^	72.91 ± 0.119 ^f^	80.51 ± 0.21 ^a^	76.84 ± 0.13 ^b^	74.32 ± 0.27 ^d^	71.31 ± 0.37 ^h^	71.46 ± 0.26 ^g^	74.76 ± 0.34 ^c^	67.98 ± 0.29 ^i^
	ΔH(J/g)	13.75 ± 0.23 ^a^	9.77 ± 0.24 ^f^	12.12 ± 0.26 ^b^	11.39 ± 0.21 ^d^	11.64 ± 0.10 ^c^	10.79 ± 0.13 ^e^	11.28 ± 0.09 ^d^	11.49 ± 0.27 ^cd^	9.42 ± 0.17 ^g^
Boiled	T_0_/°C	ND	ND	ND	ND	ND	ND	ND	ND	ND
	T_P_/°C	ND	ND	ND	ND	ND	ND	ND	ND	ND
	T_c_/°C	ND	ND	ND	ND	ND	ND	ND	ND	ND
	ΔH(J/g)	ND	ND	ND	ND	ND	ND	ND	ND	ND
Fried	T_0_/°C	56.07 ± 0.17 ^c^	45.02 ± 0.24 ^h^	55.14 ± 0.25 ^d^	54.72 ± 0.31 ^e^	54.35 ± 0.28 ^f^	54.05 ± 0.27 ^g^	58.23 ± 0.24 ^a^	56.97 ± 0.31 ^b^	56.19 ± 0.14 ^c^
	T_P_/°C	60.04 ± 0.34 ^e^	57.72 ± 0.19 ^g^	65.59 ± 0.12 ^a^	64.95 ± 0.15 ^b^	63.77 ± 0.17 ^c^	57.4 ± 0.12 ^g^	58.05 ± 0.24 ^f^	61.77 ± 0.27 ^d^	56.44 ± 0.26 ^h^
	T_c_/°C	64.33 ± 0.19 ^d^	68.41 ± 0.35 ^a^	65.67 ± 0.49 ^b^	64.41 ± 0.34 ^d^	61.42 ± 0.13 ^f^	61.53 ± 0.16 ^f^	65.59 ± 0.31 ^b^	64.99 ± 0.51 ^c^	62.92 ± 0.28 ^e^
	ΔH(J/g)	11.42 ± 0.11 ^a^	6.56 ± 0.14 ^d^	11.49 ± 0.13 ^a^	7.62 ± 0.13 ^c^	8.25 ± 0.16 ^b^	6.27 ± 0.18 ^e^	7.49 ± 0.21 ^c^	7.64 ± 0.15 ^c^	6.13 ± 0.17 ^f^
Microwaved	T_0_/°C	64.36 ± 0.34 ^e^	69.61 ± 0.21 ^c^	70.12 ± 0.16 ^b^	69.9 ± 0.23 ^c^	66.51 ± 0.17 ^d^	70.29 ± 0.12 ^a^	66.41 ± 0.17 ^d^	64.21 ± 0.28 ^e^	64.01 ± 0.35 ^f^
	T_P_/°C	74.31 ± 0.34 ^d^	73.84 ± 0.28 ^d^	77.19 ± 0.51 ^a^	77.42 ± 0.34 ^a^	75.14 ± 0.24 ^c^	71.47 ± 0.27 ^e^	70.58 ± 0.57 ^f^	76.21 ± 0.31 ^b^	67.23 ± 0.36 ^g^
	T_c_/°C	80.65 ± 0.46 ^d^	79.59 ± 0.24 ^e^	80.17 ± 0.51 ^d^	83.24 ± 0.28 ^c^	76.99 ± 0.34 ^g^	84.37 ± 0.54 ^a^	77.65 ± 0.27 ^f^	83.4 ± 0.26 ^b^	74.37 ± 0.29 ^g^
	ΔH(J/g)	15.98 ± 0.18 ^a^	11.75 ± 0.35 ^e^	13.48 ± 0.26 ^b^	11.58 ± 0.34 ^ef^	12.91 ± 0.38 ^c^	11.41 ± 0.21 ^f^	11.59 ± 0.21 ^ef^	12.44 ± 0.27 ^d^	11.47 ± 0.37 ^f^

Different subscript letters in the same row for the same item indicate significant differences (*p* < 0.05).

## Data Availability

The original contributions presented in the study are included in the article, further inquiries can be directed to the corresponding author.
